# Therapies for Parkinson’s disease and the gut microbiome: evidence for bidirectional connection

**DOI:** 10.3389/fnagi.2023.1151850

**Published:** 2023-05-30

**Authors:** Grace Hey, Navya Nair, Emily Klann, Anjela Gurrala, Delaram Safarpour, Volker Mai, Adolfo Ramirez-Zamora, Vinata Vedam-Mai

**Affiliations:** ^1^University of Florida, Department of Neurology and Fixel Institute, Gainesville, FL, United States; ^2^University of Florida, Department of Epidemiology and Emerging Pathogens Institute, Gainesville, FL, United States; ^3^Oregon Health and Science University, Department of Neurology, Portland, OR, United States

**Keywords:** Parkinson’s, microbiota, levodopa, deep brain stimualtion, gut brain axis, vagus nerve, short chain fatty acids

## Abstract

The gut brain axis (GBA), a bidirectional communication pathway has often been linked to health and disease, and gut microbiota (GM), a key component of this pathway shown to be altered in Parkinson’s disease (PD), are suggested to contribute to the pathogenesis of PD. There are few studies that report the impact of oral medication therapy on GM, however, there are even fewer studies that discuss the impact of other treatments such as device assisted therapies (DAT) including deep brain stimulation (DBS), levodopa-carbidopa intestinal gel infusion (LCIG) and photobiomodulation (PBM) and how these might impact GM. Here, we review the literature and summarize findings of the potential contributions of GM to the heterogenous clinical response to pharmaceutical therapies among individuals with PD. We also discuss the potential interactions between the GM and DATs such as DBS and LCIG and present evidence for alterations in GM in response to DATs. Given the complexity and highly individual nature of the GM of patients with PD and the potential influence that other external factors such as diet, lifestyle, medications, stage of the disease and other comorbidities, further investigations into the response of GM to therapies are worthy of future study in prospective, controlled trials as well as medication naïve individuals. Such detailed studies will help us further comprehend the relationship between GM in PD patients, and will help investigate the potential of targeting GM associated changes as a treatment avenue for PD.

## Introduction

Parkinson’s disease (PD) is a chronic neurodegenerative disease characterized by the loss of nigrostriatal dopaminergic innervation. The pathological hallmark is the deposition of intracellular α-synuclein (*α*-syn) aggregates in the form of Lewy bodies, and the ultimate demise of neurons and subsequent development of motor symptoms such as bradykinesia, rigidity, resting tremor, postural instability, and dystonia. It is becoming increasingly evident that non-motor symptoms including gastrointestinal (GI) dysfunction, psychiatric disturbances, sleep issues, sensory issues, and autonomy dysfunction (DeMaagd and Philip, 2015) are highly prevalent and problematic aspects of the disease. Dysregulation of the glutamatergic, cholinergic, serotonergic, and adrenergic systems are implicated in the pathogenesis of PD and contribute to heterogenous patient presentations (DeMaagd and Philip, 2015; Chaumont-Dubel et al., 2020). Treatment strategies for PD typically utilize dopaminergic medications with the goal of increasing synaptic dopamine concentrations or dopamine receptors stimulation. In conjunction with pharmacological treatments, rehabilitation interventions, including physical and speech therapy and dietary modifications are employed to alleviate both motor and non-motor symptoms of the disease. Treatment strategies for PD typically utilize levodopa regimes in attempts to replace dopamine however, disease progression ultimately results in decreased medication efficacy as well as an increased prevalence of debilitating side effects in the long term. Device-assisted therapies (DATs) such as deep brain stimulation (DBS) (Limousin-Dowsey et al., 1999), levodopa-carbidopa intestinal gel infusion (LCIG) (Senek and Nyholm, 2014; Olanow et al., 2020) and subcutaneous apomorphine injections (Chaudhuri et al., 1988; Stibe et al., 1988; Pollak et al., 1989) are recommended for some PD patients with motor fluctuations and tremors inadequately controlled by medication, or to those patients who are intolerant to medications. Other therapies such as transcranial magnetic stimulation (TMS) (Pascual-Leone et al., 1994; Valls-Solé et al., 1994) and photobiomodulation (PBM) (Santos et al., 2019) have also been explored as potential alternatives to medications. The goal of this review is to provide a brief overview of the existing literature in the context of GM and PD and how GM could influence PD treatment outcomes, with a specific focus on the influence that DATs might have on GM, which has not yet been systematically investigated. We also provide a brief summary of key studies on GM and PBM and acknowledge that discussions relating to apomorphine injections, and TMS are out of the scope of this review.

### Gastrointestinal symptoms in patients with PD

Gastrointestinal (GI) issues are typically observed in almost all stages of PD, and about 30% of PD patients complain of GI symptoms, such as constipation, drooling, dysphagia, and gastroparesis (Pfeiffer, 2003). One of the most common prodromal symptoms of PD is constipation (Abbott et al., 2001; Savica et al., 2009; Lin et al., 2014), and is also the most prevalent GI symptom (8 to 70%) in patients with PD (Edwards et al., 1991; Knudsen et al., 2017; Stocchi and Torti, 2017). Despite extensive research, the etiology of constipation and whether constipation in PD is caused by gut or brain pathology remains elusive (Borghammer, 2018). One case–control study reported an elevated PD risk among participants with a history of constipation, as early as 20 years before onset of the first motor symptoms, as assessed by medical record review (Savica et al., 2009). Drooling is related to swallowing dysfunction during the oropharyngeal phase (Srivanitchapoom et al., 2014) and increased in parotid gland secretion (Nicaretta et al., 2008) that is only compounded by flexed posture, unintended and open mouth (Kalf et al., 2011). The prevalence of dysphagia is fairly high (97%) in objective studies (Pfeiffer, 2018) and is seen in patients with advanced PD with severe bradykinesia and rigidity, which likely contributes to oropharyngeal dysphagia (Fasano et al., 2015). The prevalence of gastroparesis is very high (70 to 100%), and even though the underlying pathophysiology is unclear, plays a major player in the development of motor fluctuations in PD (Doi et al., 2012). The average half-emptying time in patients with mild PD is 46 to 149 min, and is 55 to 221 min in moderate/severe PD, whilst the average half-emptying time in healthy controls is to 43 to 107 min (Heetun and Quigley, 2012).

### Evidence of leaky gut and bowel inflammation in PD

In a rat model of PD where substantia nigra inflammation and selective dopaminergic neuronal loss were induced by LPS injection, it was shown that bowel inflammation can exacerbate neuroinflammation, has the ability to disrupt the blood brain barrier (BBB), and can result in dopaminergic neuronal loss in the substantia nigra (Villarán et al., 2010). Subsequently, some studies have shown clinical evidence of bowel inflammation in PD patients for example; Devos et al. found increased expression of proinflammatory cytokines (tumor necrosis factor alpha (TNF-*α*), interferon gamma (IFN-*γ*), interleukin (IL)-6, and IL-1*β*) in PD patients using real-time PCR analysis of mRNA expression of pro-inflammatory cytokines in the ascending colon biopsies of PD patients and controls (Devos et al., 2013). They report this increase to correlate with increased expression of glial markers (glial fibrillary acidic protein and Sox-10), suggesting that proinflammatory events in the bowel are increased in PD patients. Notably, the authors report that expression levels of cytokines and glial markers did not correlate with immunostaining levels of phospho-α-synuclein or axial symptom sub-scores on the UPDRS part III. Further, these expression levels correlated negatively with PD duration, suggesting that bowel inflammation may play a role in PD pathogenesis (Devos et al., 2013). Consistent with this hypothesis, immune profiles in the stool of PD patients show elevated levels in proteins related to angiogenesis and chemokines and cytokines such as IL-1α, IL-1*β*, and IL-8 when compared to controls (Houser et al., 2018). Results from another study also shows increased fecal calprotectin, an intestinal permeability marker in patients with PD compared to age-matched controls yet, the level of fecal calprotectin did not correlate with clinical parameters such as disease duration (Schwiertz et al., 2018; Dumitrescu et al., 2021).

There is evidence from recent studies suggesting that gut inflammation in PD can be traced to increased intestinal permeability, or leaky gut which correlates with intestinal α-synuclein accumulation in patients with PD (Forsyth et al., 2011). They also observed increased urine sucralose excretion in patients with PD when compared to controls, suggestive of increased colonic permeability (Forsyth et al., 2011). Greater colon permeability has shown to correlate with increased α-synuclein accumulation and *E. coli* in distal sigmoid biopsies of PD patients (Salat-Foix et al., 2012). Hence, increased permeability-related bowel inflammation and an increased chance of gut bacteria translocation are likely involved in PD pathogenesis.

Examination of morphological changes and expression in the intestinal epithelial barrier (including two tight junction proteins, ZO-1 and occludin) in colonic biopsy tissues of PD patients and controls showed that a greater proportion of PD patients had disrupted and irregularly distributed tight junction proteins and lower expression levels of occludin compared to controls (Clairembault et al., 2015) thus confirming observations of increased gut permeability and mild bowel inflammatory changes in patients with PD.

## Brain-gut communication

It is now known that there is extensive bidirectional communication between the brain and the gut *via* several immune, endocrine, metabolic and neural pathways commonly referred to as the gut brain axis (GBA) central to which are the GM (Carabotti et al., 2015), resulting in a nexus that is often referred to as the microbiota-gut-brain axis (Gershon, 1999; Rhee et al., 2009; Round and Mazmanian, 2009; Forsythe et al., 2014; Mulak and Bonaz, 2015; Reigstad et al., 2015; de Vadder et al., 2018; Cryan et al., 2019; Osadchiy et al., 2019). This complex, intricate colonization has co-evolved over thousands of years to form a mutually beneficial relationship (Backhed et al., 2005; Neish, 2009). The nutrient-rich environment of the gut provides for the collection of bacteria, archaea and eukarya. GM can impact the host through the production of various metabolites including short-chain fatty acids (SCFAs), cytokines, hormones and neurotransmitter precursors (Dethlefsen et al., 2007; Carabotti et al., 2015). GM can also influence several physiological functions including improving gut integrity, adapting of intestinal epithelia (Natividad and Verdu, 2013), effecting host metabolism *via* SCFAs (den Besten et al., 2013), conferring protection against pathogens (Baumler and Sperandio, 2016) and modulating the host immune system (Gensollen et al., 2016). The vagus nerve is able to sense microbiota, and transfer this information to the CNS where it is integrated and to then generate a response (Eisenstein, 2016; Tse, 2017). For more detailed descriptions on the role of the vagus in the GBA which is not the primary focus of this review, the reader is referred to several reviews including a recent review from our group (Fulling et al., 2019; Klann et al., 2021; Bonaz, 2022; Bostick et al., 2022; Dicks, 2022; Han et al., 2022; Raj et al., 2022; Tan et al., 2022).

### Gastric *Helicobacter pylori* and small intestinal microbial overgrowth

*Helicobacter pylori* infection is a common, chronic infection and has been implicated in PD (Charlett et al., 1999; Dobbs et al., 2010; Nielsen et al., 2012; Blaecher et al., 2013; Efthymiou et al., 2017), with epidemiological studies showing an increased risk of *H. pylori* infection in PD. Meta-analyses have also found a significantly worse mean UPDRS score in PD patients with *H. pylori* infection (either UPDRS or total UPDRS III in “on” or “off” state) (Tan et al., 2015; Dardiotis et al., 2018) and an improvement in UPDRS part III scores after *H. pylori* has been treated (Hashim et al., 2014; Tan et al., 2015). While mechanisms of involvement of chronic *H. pylori* infection in PD are unclear, they can be likely traced to multiple factors such as *H. pylori* toxin, neuroinflammation, and gut microbiota alterations (McGee et al., 2018).

Data from cross-sectional studies show that small intestinal bacterial overgrowth (SIBO) is more prevalent in patients with PD than in healthy controls (prevalence of 25 to 54%) (Gabrielli et al., 2011; Niu et al., 2016; DiBaise et al., 2018), and the data on the relationship between PD symptoms and SIBO is quite heterogeneous (Fasano et al., 2013). However, rectification of SIBO has shown to improve motor fluctuations in PD patients, despite the high recurrence rate at 6 months (43%) (Fasano et al., 2013).

### Gut microbiota alterations In PD

Given the extent of physiological signaling between the brain gut axis involving microbiota, it is not surprising that its dysfunction has been attributed as a cause for many neurological disorders (Sekirov et al., 2010).

This is notably true in PD (Bullich et al., 2019). There are now over 20 comprehensive studies that have investigated PD associated differences in gut microbiota composition (Hasegawa et al., 2015; Scheperjans et al., 2015; Unger et al., 2016; Li et al., 2017; Heintz-Buschart et al., 2018; Lin et al., 2018). These reports suggest that α-diversity (species diversity within a single subject) is similar in most datasets, and all studies showed differences in microbiota composition between PD patients and controls (*β*-diversity), but the actual bacteria were heterogeneous. For instance, compared to controls, the family Verrucomicrobiaceae (phylum Verrucomicrobia) and genera *Akkermansia* (phylum Verrucomicrobia; family Verrucomicrobiaceae) and *Lactobacillus* (phylum Firmicutes; family Lactobacillaceae) were seen to be overrepresented in PD patients in several studies (Scheperjans et al., 2015; Bedarf et al., 2017; Hill-Burns et al., 2017; Heintz-Buschart et al., 2018). However, the families Prevotellaceae (phylum Bacteroidetes), Lachnospiraceae (phylum Firmicutes), and Pasteurellaceae (phylum proteobacteria) and genera *Blautia* (phylum Firmicutes; family Lachnospiraceae), *Roseburia* (phylum Firmicutes; family Lachnospiraceae), *Prevotella* (phylum Bacteroidetes; family Prevotellaceae), and *Faecalibacterium* (phylum Firmicutes; family Clostridiaceae) were seen to be underrepresented in PD patients in some studies (Scheperjans et al., 2015; Unger et al., 2016; Bedarf et al., 2017). *Prevotella* is observed abundantly in people who consume primarily plant-based, fiber-rich diets, which act as substrates for bacteria for the production of SCFAs (Ley, 2016). *Prevotella* is a mucin-degrader that promotes and serves as an indicator of gut integrity (Brown et al., 2011) nevertheless, it has also been linked to gut inflammation and systemic inflammatory conditions (Ganesh et al., 2013; Ley, 2016).

Delineating the relationship between fecal microbiota and PD clinical features has resulted in a mixed bag. Two studies observed a correlation of PD duration with the abundance of *Escherichia/Shigella* genera, or family Enterobacteriaceae among other nonoverlapping bacteria (Li et al., 2017; Petrov et al., 2017), and the abundances of Enterobacteriaceae were also shown to correlate with severity of postural instability and gait disturbance in another report (Scheperjans et al., 2015).

### Anti-PD medications and gut microbiota

While the primary focus of this review is DATs and GM modifications, it is pertinent to discuss other therapies in this context. The influence between GM and drug intake is mutual, and is well reported in the literature (Li et al., 2016; Collins and Patterson, 2020; Vich Vila et al., 2020) with a growing body of evidence emerging on the relationship between commonly prescribed PD drugs and GM (Jameson and Hsiao, 2019; Maini Rekdal et al., 2019; van Kessel et al., 2019; Zhang et al., 2021). Levodopa (L-dopa), the dopamine precursor and the gold standard for the treatment of PD (Salat and Tolosa, 2013) is usually taken in combination with a dopa decarboxylase inhibitor, such as carbidopa to prevent early conversion into dopamine prior to reaching the brain (van Kessel et al., 2019). Yet, carbidopa is ineffective against the bacterial dopa decarboxylases (van Kessel et al., 2019), which enables GM to metabolize L-dopa, into dopamine by a dopa decarboxylase from *E. faecalis* and then converted into m-tyramine through the action of a dehydroxylase from *Eggerthella lenta*, thus decreasing drug availability and increasing side effects (Jameson and Hsiao, 2019; Maini Rekdal et al., 2019; van Kessel et al., 2019). Deamination of L-dopa by *C. sporogenes* results in 3-(3,4-dihydroxyphenyl) propionic acid, relatively high levels of which have been reported in feces of L-dopa treated PD patients (van Kessel et al., 2019).

L-dopa metabolism is dependent on intestinal bacteria but in addition, it influences the GM composition itself based on reports that show an increased relative abundance of *Peptoniphilus*, *Finegoldia* and *Enterococcus*, and a decrease in *Faecalibacterium*, *Blautia* and *Lachnospirae* after L-dopa therapy (Weis et al., 2019; van Kessel et al., 2020).

Catechol-o-methyl transferase (COMT) inhibitors, anticholinergics, monoaminoxidase inhibitors and dopamine agonists are all additional PD drugs that can be administered with or without L-dopa (Weis et al., 2019), and they can impact the abundance of gut microbial dopa decarboxylases, thus influencing dopamine metabolism (van Kessel et al., 2019). Dopamine agonist therapy has been associated with reduced intestinal motility and SIBO in a rat model (van Kessel et al., 2022), and the authors proposed these effects to be mediated by greater relative abundance of *Lactobacillus* and *Bifidobacterium*, along with a concurrent decrease in *Lachnospiraceae* and *Prevotellaceae* (van Kessel et al., 2022). There are known GI side effects induced by COMT inhibitors and anticholinergics (Kaakkola, 2000; Ness et al., 2006; Gray et al., 2022), likely due to gut dysbiosis (Chen et al., 2021). Some studies have shown gut microbial signatures such as increased *Bifidobacterium* or *Lactobacillaceae* in PD patients treated with COMT inhibitors (Scheperjans et al., 2015; Hill-Burns et al., 2017; Aho et al., 2019). Further, a significant decrease was observed in the abundance of *Faecalibacterium prausnitzii* along with lowering of fecal butyrate associated with a widely prescribed COMT inhibitor (entacapone) (Gordin et al., 2004). Several other studies have confirmed that there is an alteration of GM related to GI disorders and constipation after entacapone treatment is initiated (Weis et al., 2019; Fu et al., 2022). Specifically, Fu et al. showed that there is a downward trend in *Sellimonas*, *Lactobacillus*, *Faecalibacterium*, *Dorea*, *Intestinobacter* and *Blautia* and an upwards trend in *Eubacterium*, *Bifodobacterium* and *Christensenellacea* R-7 group in a group of PD patients receiving entacapone in combination with L-dopa when compared to those treated with L-dopa alone (Fu et al., 2022).

These findings lend support to the hypothesis that there is an important relationship between microbiota and drug metabolism through the disease course of PD patients and hence systematic profiling of the GM will be essential to comprehend mechanistic underpinnings of GM–drug interactions and how this interplay affects drug efficacy. Additional studies are needed to assess the potential utility of therapeutics in altering the GM to enhance therapeutic efficacy and clinical outcomes in patients with PD.

Thus overall, there is sufficient support for the concept of a bidirectional communication along the brain-gut-microbiome-immune axis (Singh et al., 2016; Galley et al., 2017; Karl et al., 2018; Jacobs et al., 2021), which can play a crucial role in PD. While there is some evidence for GM in PD medications as discussed previously, there is very little available in the literature regarding the effects of DATs on the GBA in the context of PD. The remainder of the review will focus on DATs in PD, and will discuss a few non-DATs useful in treating PD in the context of GM.

## Device-assisted therapies for PD

While traditional therapies such as L-dopa are effective for the treatment of PD motor symptoms, long-term use of such medications, disease progression and severity can lead to the development of motor complications and potential psychiatric symptoms (Nakamura, 1997; Olanow et al., 2014). This can be attributed to oral levodopa’s short plasma half-life, which results in pulsatile striatal receptor simulation and consequently, adverse side-effects (Shoulson et al., 1975; Nutt, 2008). When oral pharmaceutical treatment strategies become less effective, PD patients can be considered for DATs. Several DATs have demonstrated efficacy in PD patients and can improve motor fluctuations, including deep brain stimulation (DBS), levodopa-carbidopa intestinal gel infusion (LCIG), and continuous subcutaneous apomorphine injections (Marsili et al., 2021).

DBS was originally approved by the Food and Drug Administration in 2002 and has since become a relatively commonly utilized therapy for the treatment of advanced PD in many developed nations, although it is significantly under-utilized in many parts of the world (Lim et al., 2019). During surgery, a quadripolar deep-brain stimulation electrode is stereotactically inserted into the brain through a burr hole in the skull connected to an implantable pulse generator (Okun, 2012). The subthalamic nucleus (STN) and globus pallidus interna (GPi) are commonly targeted structures in DBS for PD patients. Other emerging targets include ventralis intermedius (Vim) DBS, which has shown to improve tremor symptoms. Further evidence demonstrates variable improvement of cognition (which can also be negatively impacted by DBS especially in individuals with pre-existing cognitive impairment (Bucur and Papagno, 2022; Hamdan and Vieira, 2022)), psychiatric disturbances, mood disorders, autonomic dysfunction, and sleep issues following GPi and STN DBS for PD (Fasano et al., 2012; Fukaya and Yamamoto, 2015; Mansouri et al., 2018).

In addition to DBS, it has been hypothesized that pulsatile striatal receptor simulation seen in patients utilizing long-term traditional levodopa therapy can be negated by delivering dopamine in a continual manner. One strategy that aims to stabilize plasma levels of levodopa through continuous dopaminergic innervation is levodopa-carbidopa intestinal gel (LCIG) therapy (Olanow et al., 2014). LCIG involves the continual infusion of a carboxymethylcellulose aqueous gel to the proximal jejunum through a percutaneous gastrojejunostomy tube powered by a portable infusion pump (Olanow et al., 2014). LCIG has been shown to significantly improve motor functioning in PD patients by providing reductions in “off” time (Nyholm et al., 2012; Olanow et al., 2014; de Fabregues et al., 2017; Fernandez et al., 2018). Additionally, LCIG reduces the prevalence of non-motor symptoms including psychiatric disturbances, cognitive deficits, sleep issues, and gastrointestinal dysfunction (Chang et al., 2016). Similar to LCIG, apomorphine can be continually administered *via* DAT to provide PD patients with continuous dopaminergic stimulation and consequently, motor and non-motor symptom relief. Apomorphine is a non-narcotic derivative of morphine that activates striatopallidal pathways *via* direct stimulation of postsynaptic D1 and D2 striatal dopamine receptors (Hagell and Odin, 2001; Carbone et al., 2019). The structure, composition, and lipophilic nature of apomorphine allows it to cross the blood–brain barrier more readily than levodopa (Carbone et al., 2019). For this reason, apomorphine is commonly used as a “rescue medication” due to its fast-acting pharmacokinetic properties (Hagell and Odin, 2001; Carbone et al., 2019). When used as a “rescue medication,” the effects of apomorphine onset approximately 4–12 min and last approximately 45–60 min after the time of injection (Hagell and Odin, 2001; Carbone et al., 2019). However, regularly-scheduled injections of apomorphine can provide long-term PD symptom relief. Intermittent injections of apomorphine can be administered into the abdomen, arms, or thighs with pre-marked doses contained in an insulin syringe mounted in an injector pen (Hagell and Odin, 2001). If injections are needed more than 4–6 times daily, continuous apomorphine infusions can be administered through an infusion pump (Hagell and Odin, 2001). All DATs need to be carefully considered in suitability to patient profiles and co-morbidities, as well as permissible potential side effect profiles from each DAT (Hagell and Odin, 2001; Carbone et al., 2019).

## Interactions between the gut microbiome and device-assisted therapies

The utilization of DATs for the treatment of PD has significant implications on the GM. As discussed in previous sections, compared to healthy, non-PD controls (HCs), PD patients have distinctly different GM. Patients have been shown to have significant overrepresentation of *Verrucomicrobia* at the phylum level, *Verrucomicrobiales* at the order level, *Verrucomicrobiaceae* and *Lactobacillaceae* at the family level, and *Akkermansia* at the genus level (Lubomski et al., 2022a,b). Similarly, *Firmicutes* and *Bacteroidetes* at the phylum level, *Pasteurellales* at the order level, *Pasteurellaceae, Butyricicoccaceae*, and *Veillonellaceae* at the family level, *Blautia, Faecalibacterium, Roseburia, Fusicatenibacter, Haemophilus, Gemmiger, Lachnospiraceae ND3007 group, Erysipelotrichaceae, Butyricicoccus* and *Streptococcus* at the genus level are underrepresented in PD patients when compared to non-PD HCs (Lubomski et al., 2022a,b). Prevalence of such diverse GM dysregulations in PD exemplifies the significant role of the GM in PD pathology. The composition of the GM is further altered following the initiation of DATs, specifically DBS and LCIG. It is important to note that reports investigating the interactions between the GM and DATs are relatively limited (Melis et al., 2021; Lubomski et al., 2022a,b). There are no reports in the literature describing the relationships between intermittent or continual apomorphine infusions and the GM (Table 1).

**Table 1:** Overview of studies investigating the effect of DAT activation and PBM on the composition of the GM.

Source	Intervention	Conclusion
Lubomski et al. (2022b)	4 weeks DBS	Significant differences in alpha and beta diversity were seen when compared to initiation of LCIG therapy (*p* = 0.0102)
		Use of the post-operative antibiotic Cephazolin may have caused increased taxa differences
		Overabundance of *Parabacteroides* coupled with *Clostridium* could serve as an anti-inflammatory mechanism to promote post-operative healing
Lubomski et al. (2022a)	12 months DBS	Increased longitudinal diversity could be due to time-dependent changes in the GM secondary to DAT activation
Lubomski et al. (2022b)	4 weeks LCIG	Significant differences in alpha and beta diversity were seen when compared to initiation of DBS (*p* = 0.0102)
		Overexpression of *Escherichia/Shigella* may be attributed to the acidic properties of LCIG gel
		First reported under-expression of *Gemmiger* highlighting PD-specific GM alterations secondary to DAT activation
Lubomski et al. (2022a)	12 months LCIG	Increased longitudinal diversity could be due to time-dependent changes in the GM secondary to DAT activation
Melis et al. (2021)	LCIG	Administration of LCIG therapy significantly increased the abundance of *Enterobacteriaceae*, *Escherichia* and *Serratia* compared to administration of levodopa alone
		Increased abundance of *Enterobacteriaceae* may contribute to gut inflammation following LCIG initiation
Bicknell et al. (2022)	12 weeks PBM	Decreased F:B ratio was observed which is indicative of improved gut health
		Increase in SCFA-producing bacteria was observed

One study investigating the role of acute DAT use (either DBS or LCIG) on the GM composition (at pre-therapy time points of −2 and 0 weeks with post-therapy time points of +2 and + 4 weeks to healthy controls at 0 weeks) revealed significantly greater (*p* = 0.0033) phylogenetic GM abundances following DAT initiation (Lubomski et al., 2022b). These differences were seen consistently at different timepoints for the same individuals (Lubomski et al., 2022b). Although alpha diversity measures including Shannon (species abundance and evenness within a community) and Simpson (species richness and evenness within a community) scores were not significant between DAT and non-PD HC groups, measurements of beta diversity (extent of difference in species diversity difference between two environments) were significant between DAT groups (*p* = 0.0102) (Lubomski et al., 2022b). Specifically following DBS, *Clostridium_XlVa, Bilophila, Parabacteroides* and *Pseudoflavonifractor* were overrepresented while *Dorea* was underrepresented. Greater taxa differences in this group may be due to postoperative administration of Cephazolin, an antibiotic utilized to prevent DBS hardware infections (Lubomski et al., 2022a,b). However, the symbiotic overabundance of *Parabacteroides* coupled with *Clostridium* could serve as an anti-inflammatory mechanism, as both species produce bile acids (Lubomski et al., 2022a,b). Following acute administration of LCIG therapy, *Pseudoflavonifractor* was overrepresented while *Escherichia/Shigella* and *Gemmiger* were underrepresented (Lubomski et al., 2022b). The authors proposed *Escherichia/Shigella* overexpression may have been due to the mildly acidic (~ pH 6.0) properties of the LCIG gel itself as *Escherichia/Shigella* tolerate acidic conditions (Benjamin and Datta, 1995; Lubomski et al., 2022b). This study reported the underrepresentation of *Gemmiger* for the first time, highlighting how DATs induce GM alterations specific to PD (Lubomski et al., 2022b).

**Figure 1:**
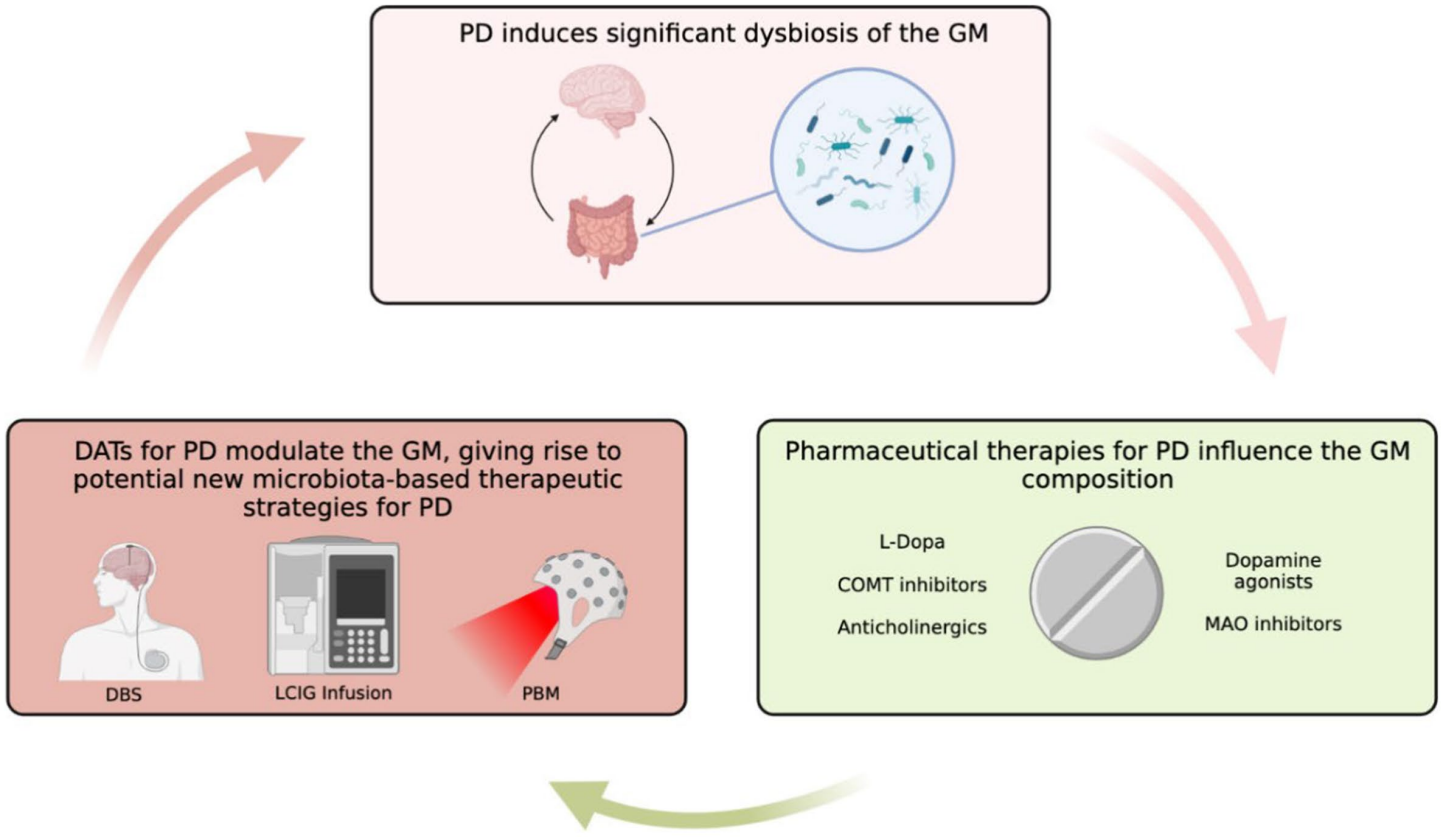
Overview of GM in PD and the role that DATs might have in influencing PD-GM. Created with BioRender.com.

Interestingly, a similar study investigating the role of longitudinal (0 month–12 month) use of DATs on the GM composition revealed similar, yet important differences. Both DBS and LCIG therapy elicited specific changes in the GM composition. However, the short-term taxa abundance changes reported by Lubomski et al. (2022a) were inconsistent with longitudinal results (Lubomski et al., 2022a). For example, long-term exposure to DBS resulted in the overrepresentation of *Euryarchaeota* and *Spirochaetes* at the phylum level, *Bacillales, Methanobacteriales*, and *Spirochaetales* at the order level, *Methanobacteriaceae, Bacillaceae*, and *Spirochaetaceae* at the family level, and *Prevotella*, *Methanobrevibacter, Treponema, Bacillus, Veillonella, Citrobacter, Faecalicoccus*, and *Morganella* at the genus level (Lubomski et al., 2022a). *Hespellia, Acetanaerobacterium, Anaerotruncus, Howardella*, and *Flavonifractor* were underrepresented following long-term DBS exposure (Lubomski et al., 2022a). Similarly, long-term LCIG therapy resulted in overrepresentation of *Prevotellaceae, Roseburia, Prevotella*, and *Bacillus* (Lubomski et al., 2022a). *Hespellia, Eggerthella, Holdemania, Gordonibacter*, and *Acetanaerobacterium* were underrepresented following long-term LCIG use (Lubomski et al., 2022a). Treatment formulation could also result in alterations in bacterial abundance. For instance, patients receiving an L-dopa + carbidopa intestinal gel showed higher levels of *Enterobacteriaceae*, *Escherichia* and *Serratia* compared to those receiving only L-dopa, and both groups displayed metabolic markers of gut inflammation (Melis et al., 2021). Contrastingly, results from another study conducted on 19 PD patients prior to, and after a 90-day L-dopa treatment reported no major differences in either α or β diversity between the two time points, which indicates that more research in this area is needed to better understand factors implicated in L-dopa-mediated GM reshaping (Palacios et al., 2021). The observed differences between acute and longitudinal DAT use on the microbiota integrity and composition highlight how GM modifications may emerge and evolve in a time-dependent manner. Although the mechanisms are still not fully understood, this could be due to evolving physiological responses to DATs or DAT-mediated influences on the GM that emerge as exposure time to DATs increases (Lubomski et al., 2022a). Furthermore, it is likely that the interactions between the GM and DATs is bidirectional given the significant modulatory effects of the GM on other organs and physiological processes (Figure 1). However, this remains an area of limited investigation, hence future long-term, longitudinal studies in this area will be of interest.

### Other therapies

#### Photobiomodulation

Photobiomodulation or PBM therapy uses non-thermal and non-ionizing light in the visible and infrared spectrum, and has recently been proposed as a therapeutic intervention for symptomatic improvement in PD of patients with Parkinson’s disease (Johnstone et al., 2015). PBM is thought to target cytochrome-C-oxidase, which absorbs red and near-infrared light (Hamblin, 2018), which in turn releases reactive oxygen species (ROS) from the complex, thereby promoting increased mitochondrial membrane potential. This leads to an increase in ATP production, which can regulate downstream signaling pathways and gene transcription *via* ATP, cAMP, ROS, Ca^2+^ and nitric oxide (NO) (Hamblin, 2018; Benson et al., 2020). Data of PBM from preclinical models of PD are promising, showing the ability to protect animals when PBM delivered to areas remote from the brain prior to administration of MPTP (pre-conditioning) (Johnstone et al., 2015; Kim et al., 2017; Ganeshan et al., 2019; Gordon and Johnstone, 2019). These data have prompted the justification of clinical trials (Salehpour and Hamblin, 2020). A recent, prospective proof-of-concept study which used both transcranial as well as remote PBM in 12 participants with PD showed that it is a safe, effective therapy for several PD symptoms as long as the treatment was continued (Liebert et al., 2021). Data from this study is encouraging, and warrants larger randomized clinical trials.

#### Photobiomodulation of the gut microbiome

Bicknell et al. showed that PBM when applied to mice abdomen can lead to alterations in GM (Bicknell et al., 2019). Furthermore, recent reports suggest that in humans, combination PBM therapy when delivered to the head, nose, neck and abdomen has the potential to attenuate or reverse some of the clinical signs and non-motor symptoms of PD (Santos et al., 2019). A recent study compared fecal microbiome samples (pre- and post-treatment) from PD patients before and after a 12-week course of PBM therapy to the abdominal, head, neck and nasal areas (Bicknell et al., 2022). Results from this study show that at the phylum level, there is a decrease in Firmicutes, and an increase in Bacteroidetes, resulting in a “positive” alteration in the Firmicutes to Bacteroidetes (F:B) ratio (Eckburg et al., 2005). A higher ratio is often reported to be an indicator of poor gut health and is often used as a proxy for gut health (Ley et al., 2006; Spychala et al., 2018; Magne et al., 2020; Stojanov et al., 2020). Microbial diversity was not significantly altered after PBM treatment, however, there was an increase in SCFA-producers, and in those genera generally recognized to be beneficial and a decrease in potential pathogens (Bicknell et al., 2022). Further in-depth investigations into the response of GM to PBM is worthy of prospective, controlled clinical trials which could provide insight into the potential of targeting the gut microbiome with PBM as an avenue into the treatment of PD.

## Discussion and future directions

Overall, our review summarizes the link between dysbiosis and PD, with a particular focus DATs, as the knowledge gleaned from this is likely important not only for diagnostic purposes, but also for therapeutic applications. However, several limitations in GM studies still exist. One concern is that there are discrepancies and variabilities found between some PD-GM studies, which limit reproducibility. Hence, it is essential to adopt and share common laboratory protocols, bioinformatics pipelines and analytical methods reduce external confounders (Boertien et al., 2019). It will also be essential to compare samples from PD patients at different stages of disease progression, especially those with prodromal disease and early subjects in order to better define stage-associated signatures (Boertien et al., 2019). Furthermore, it is essential to obtain sufficient comparisons of microbial profiles between patients undergoing conventional pharmacological treatment and treatment-naïve PD individuals in order to account for drugs that may affect GM composition (Van Kessel et al., 2019; Weis et al., 2019; Melis et al., 2021). Additionally, the dose and time of administration of GM-modifying drugs may also vary depending on the stage of the disease, thus possibly further influencing the study outcomes. Optimum probiotic-prebiotic cocktails and ideal dietary interventions have yet to be identified for PD treatment.

Fecal microbial transplants (FMT) involves transplantation of fecal microbiota from healthy donors into the GI tract of recipients to modulate and restore the GM (Zhang et al., 2012). FMT has been shown to be beneficial in patients with *Clostridium difficile* infections and irritable bowel syndrome (IBS) where there is dysbiosis (Barichella et al., 2016). Presently, there are multiple ongoing clinical trials investigating the effects of FMT on PD symptoms including constipation, motor symptoms, and restoration of gut homeostasis (Xue et al., 2020; Kuai et al., 2021; Metta et al., 2021; Segal et al., 2021; DuPont et al., 2023), NCT03808389, NCT04854291, NCT05204641, NCT03671785 and it is possible that utilization of FMT in combination with DATs could improve PD progression and symptomology.

In the context of DATs for PD, it is important to note that the studies of DAT activation and GM remain limited to a few studies, or do not exist at all. For example, there are no reports in the literature detailing the relationship between longitudinal apomorphine infusions and the composition of the GM. Administration of apomorphine for PD is similar to LCIG therapy in the sense that both medications aim to increase and stabilize dopaminergic stimulation by means of a DAT that continually infuses medication. Because LCIG therapy has been shown to induce significant changes in the PD GM (Lubomski et al., 2022b), investigating the relationship between device-assisted apomorphine infusions and the GM could be b, investigating the relationship between device-assisted apomorphine infusions and the GM could be beneficial to better understand the pathogenesis of PD and potentially utilize the GM for therapeutic benefits.

Additionally, many studies investigate the effects of DAT activation on the GM in a unidirectional manner when in reality, this is likely a bidirectional process. Accumulating evidence suggest increased intestinal permeability in PD is correlated with gut inflammation and increased prevalence of gut bacterial translocation (Forsyth et al., 2011). It is possible that DAT activation could result in translocation of the GM due to increased intestinal permeability. Furthermore, alterations in the GM secondary to DAT activation have the potential to provide therapeutic benefits. For example, following DBS for PD, *Clostridium* and *Parabacteroides* are overrepresented, possibly due to postoperative administration of Cephazolin, an antibiotic utilized to prevent DBS hardware infections (Lubomski et al., 2022a¸b). However, the symbiotic overabundance of these bacteria could serve as an anti-inflammatory mechanism to promote bodily healing following surgery (Freedman et al., 2018; Lubomski et al., 2022b). While this study looked at GM composition over a 4-week time period, it would be beneficial in the future to conduct longitudinal studies that would be helpful in better understanding the potential bidirectional relationships between DBS activation and the GM. Similarly, the somewhat contradictory findings in the GM composition after 0 month – 6 month and 0 month – 12 month initiation of LCIG therapy show how the GM has the potential to be modified in a time-dependent manner following the activation of a single DAT (Lubomski et al., 2022a). As with DBS, longitudinal investigations of LCIG therapy for PD could allow for a better understanding of the potential bidirectional relationship between DAT activation and alterations in the GM composition.

Overall, though much work remains to be done and large clinical trials are required, GM is emerging as a promising diagnostic and therapeutic tool for PD and deserves further investigation.

## Author contributions

VV-M conceptualized the article, composed and integrated sections of the review, and provided overall supervision for this project. GH, NN, AG, and EK composed sections of the review. DS, VM, and AR-Z provided critical revisions of the review. All authors contributed to the article and approved the submitted version.

## Conflict of interest

The authors declare that the research was conducted in the absence of any commercial or financial relationships that could be construed as a potential conflict of interest.

## Publisher’s note

All claims expressed in this article are solely those of the authors and do not necessarily represent those of their affiliated organizations, or those of the publisher, the editors and the reviewers. Any product that may be evaluated in this article, or claim that may be made by its manufacturer, is not guaranteed or endorsed by the publisher.
